# Decomposition Reaction Mechanism of Ammonium Perchlorate on N-Doped Graphene Surfaces: A Density Functional Theory Study

**DOI:** 10.3390/molecules30040837

**Published:** 2025-02-11

**Authors:** Zihang Zhao, Chi Zhang, Xiaogang Mu, Meng Li, Yinghui Ren, Jiachen Li, Fengqi Zhao, Haixia Ma

**Affiliations:** 1 Xi’an Key Laboratory of Special Energy Materials, School of Chemical Engineering, Northwest University, Xi’an 710069, China; 202221107@stumail.nwu.edu.cn (Z.Z.); tnt61661@163.com (C.Z.); 202322086@stumail.nwu.edu.cn (M.L.); yiren@nwu.edu.cn (Y.R.); jiachen_li@nwu.edu.cn (J.L.); zhaofqi@163.com (F.Z.); 2 Zhijian Laboratory, Xi’an 710025, China; muxg2001@163.com

**Keywords:** ammonium perchlorate, nitrogen-doped graphene, density functional theory, reaction pathway

## Abstract

The detailed decomposition pathway of ammonium perchlorate (AP) is important for the design of solid propellants containing AP. In this paper, the possible decomposition reactions of AP upon nitrogen-doped graphene (N-Gr) as a catalyst are investigated via density functional theory. The reaction pathways of HClO_4_ and NH_3_ on the N-Gr surface are explored. The decomposition reaction path of the HClO_4_ molecule on the N-Gr is HClO_4_ → ${\mathrm{ClO}}_{3}^{-}$ → ${\mathrm{ClO}}_{2}^{-}$ → ClO^−^ → Cl^−^. The rate-determining step of the process is the Cl-O bond-breaking reaction of ${\mathrm{ClO}}_{2}^{-}$ anions, and the activation energy of the reaction is 0.849 eV. The oxidation of the N-Gr surface promotes the decomposition of both HClO_4_ and NH_3_. The OH groups produced during the decomposition process can promote the adsorption and decomposition of NH_3_. This work provides new insights into the decomposition of AP on N-Gr at the molecular level.

## 1. Introduction

Solid propellants are in a significant position in the field of defense. To pursue high energy, good stability, and low preparation cost, composite propellants and modified double-base propellants have become the mainstream choices in military weapons. The oxidizers, stabilizers, and combustion catalysts were added into traditional propellant formulations to improve energy performance. The oxidizer needs to provide sufficient oxygen for the combustion of other components in the composite propellants. Therefore, the oxidizer usually occupies a high proportion in propellant formulation as a main component.

Ammonium perchlorate (AP) is widely used as the oxidizer for composite solid propellants because of its low hygroscopicity and mechanical sensibility, high oxygen content, and simple production process [1,2,3]. The mass fraction of AP used in solid propellants reaches 60% to 90% [4,5,6,7], and therefore the combustion of AP has a significant influence on the combustion performance of propellants. Thus, while preparing new combustion catalysts for AP [8,9,10,11,12,13], researchers have extensively explored the decomposition mechanism of AP [14,15,16,17,18,19,20]. The decomposition process of AP is considered to be composed of two stages: low-temperature decomposition (LTD) and high-temperature decomposition (HTD) [21,22,23,24,25,26]. In the LTD stage, AP decomposes into HClO_4_ and NH_3_ molecules. Most of these two substances will be adsorbed on the surface of AP, thus preventing decomposition of AP, and the remaining HClO_4_ and NH_3_ molecules will be further decomposed. With increasing temperature, AP molecules will start to decompose and the adsorbed NH_3_ and HClO_4_ will dissociate, which is named as the HTD stage. The newly formed and desorbed NH_3_ and HClO_4_ degradation generates a variety of gas decomposition products. The addition of a catalyst regulates the electrons and the H atoms in the process, thereby changing the LTD and HTD temperatures of AP decomposition. Furthermore, various products are formed in the LTD and HTD processes, such as NH_3_ in the LTD process and O atoms in the HTD. The adsorption of these products by the catalyst has also been proved to be another mode of action in regulating AP decomposition [27,28,29]. In this process, NH_3_ undergoes decomposition through the loss of H atoms. HClO_4_, on the other hand, has two decomposition pathways, the difference being that either OH groups or O atoms are lost in the first step [30,31,32,33]. The specific decomposition reactions will be listed below.

Although graphene is recognized as a material with excellent properties, it is often unable to meet the specific needs of a particular field due to the increased demand for physicochemical properties in various industries [34]. As a way to effectively improve electronic and chemical properties, doping techniques have received great attention. Numerous studies have also been conducted on the doping methods and doping effects of heteroatoms in various carbon materials. Results have indicated that doping the surface of adsorbents with heteroatoms, functional groups, or transition metals is an effective strategy to improve their chemical activity. Among them, N-doped graphene (N-Gr) shows excellent properties, such as good electrical conductivity and high mechanical strength due to its unique structure [35,36,37]. Studies have confirmed its promising applications in the fields of electrocatalysis, photocatalysis, and sensors [38,39,40]. In the field of energetic materials [41,42,43,44], N-Gr is mostly used as a carrier for metal catalysts, which do not lead to significant changes in the AP decomposition pathway but can significantly reduce the temperature of the high-temperature decomposition stage and the total exotherm of AP. Furthermore, Hosseini, S G [43] used differential scanning calorimetry (DSC) techniques and demonstrated that nitrogen-doped graphene can decrease the HTD temperature of AP. Thus, N-Gr itself can also act as a catalyst for AP. Therefore, the decomposition mechanism of AP catalyzed by N-Gr was investigated using a theoretical calculation method.

Herein, two possible reaction paths for the decomposition of HClO_4_ were proposed. And transition state (TS) searches were performed for all the elementary reactions on these two reaction paths when different groups were adsorbed on N-Gr. The reaction heat data obtained from the TS search results were speculated to infer the reaction network for the decomposition of HClO_4_, and the activation energies were compared to discuss the possible reaction path. In addition, the sequential dehydrogenation of NH_3_ on the N-Gr surface was investigated, and the optimal reaction path was determined.

## 2. Results and Discussion

### 2.1. The Adsorption of HClO_4_ and Its Decomposition Products on the N-Gr Surface

Before investigating the specific adsorption, models of nitrogen-doped graphene and the possible C atom as the adsorption site were studied firstly. Several different C atoms are distinguished by Mulliken population analysis. The results of the population analysis and the classification of the C atoms are presented in Appendix A.

**Figure 1:**
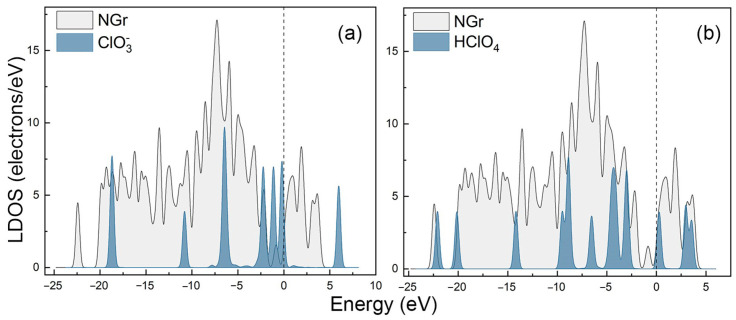
Local density of states for clean N-Gr surface and ${\mathrm{ClO}}_{3}^{-}$ (**a**); HClO_4_ (**b**) absorbed on a clean N-Gr surface.

To investigate the specific reaction mechanism, the chlorine-containing groups (HClO_x_, ${\mathrm{ClO}}_{3}^{-}$) and other groups (OH, O) were studied, respectively. The adsorption of HClO_4_ and its decomposition products on the N-Gr surface were probed, and the results of geometry optimization obtained together are presented later as reaction IS images. The results show that HClO_4_ and its decomposition products did not bond with C atoms on the N-Gr surface. In addition, the adsorption energies of HClO_x_ (x = 1~4) molecules on the N-Gr surface were calculated as −0.167~−0.031 eV, and the adsorption energies of ${\mathrm{ClO}}_{3}^{-}$, ${\mathrm{ClO}}_{2}^{-}$, and ClO^−^ on the N-Gr surface were found to be −1.214 eV, −0.183, and −0.368 eV, respectively. Clearly, the adsorption energy of ${\mathrm{ClO}}_{3}^{-}$ on the N-Gr surface was larger. In order to confirm the adsorption properties more accurately, the localized density of states (LDOS) was employed to study their interactions with N-Gr. The LDOS diagram of the HClO_4_ molecule and its decomposition products on the surface of N-Gr are shown in Figure 1.

**Figure A3:**
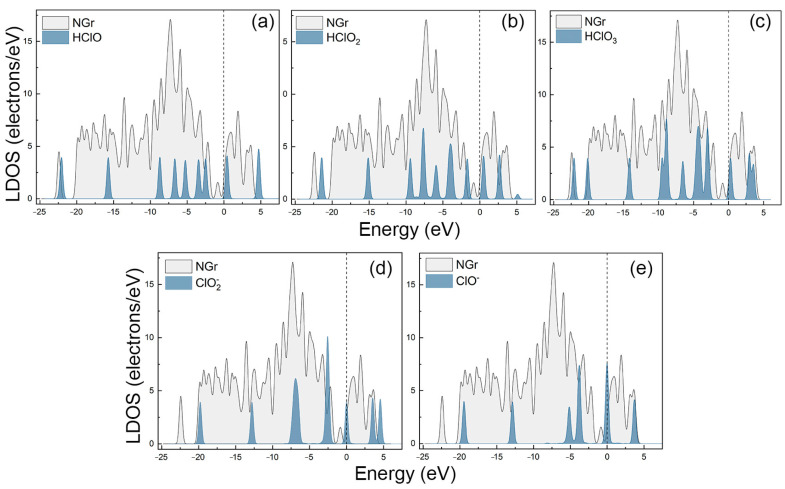
Local density of states for the molecules and acid radicals adsorbed on N-Gr surface. (**a**) HClO absorbed on a clean N-Gr surface; (**b**) HClO_2_ absorbed on a clean N-Gr surface; (**c**) HClO_3_ absorbed on a clean N-Gr surface; (**d**) ${\mathrm{ClO}}_{2}^{-}$ absorbed on a clean N-Gr surface; (**e**) ClO^−^ absorbed on a clean N-Gr surface.

Figure 1 and Figure A3 show the DOS diagram of the adsorption systems. It reveals that there is no obvious electron hybridization between the adsorbent and the N-Gr surface. Therefore, the adsorption mode of the adsorbent on the N-Gr surface is physical adsorption. It also means that the diffusion of HClO_4_ molecules and their decomposition products on the N-Gr surface occurs easily.

Considering that HClO_4_ molecules would decompose O atoms and OH groups [27,29], the adsorption of O atoms and OH groups on the N-Gr surface was also investigated. Prior to this, Mulliken population analyses showed that the N atom was surrounded by C atoms with three different charge states. They are named separately as ortho-C (o-C), meta-C (m-C) and para (p-C), and they are used as adsorption sites. The adsorption energies of O and OH at different sites and the covalent bonding lengths with the atoms on the N-Gr surface are listed in Table 1.

When O atoms are adsorbed on the top sites of m-C and p-C atoms, they tend to get transferred to the bridge sites. However, the doping of N atoms leads to the O atoms getting absorbed at the top sites of o-C atoms, and the absolute value of the adsorption energy of Oad atoms is the largest at −3.442 eV. O atoms were also able to adsorb at the top positions of N atoms; yet, the absolute value of the adsorption energy is the smallest among all stable adsorption positions on the N-Gr surface.

In contrast, OH groups which adsorbed at the bridge and cavity position would transfer to the top position or undergo C-O interruption and physically adsorb on the N-Gr surface. Moreover, OH adsorbed at the top site of the N atom would transfer to the top site of the neighboring o-C atom; in this circumstance, the adsorption energy of OHad on the N-Gr surface was the maximum at −2.393 eV. Therefore, the O atoms and OH groups generated by the dissociation were set to be adsorbed at the top site of the o-C atom in the subsequent parts.

### 2.2. Reaction Pathway for the First Step of Losing Hydroxyl Group (Acid Root Path)

Former studies specified that the acid-root path is the main decomposition path of the HClO_4_ [30,31]. Hence, the individual reactions along the acid-root path of HClO_4_ on the N-Gr surface were firstly investigated. This reaction path is shown below and marked as route A.

Route A:

$${\mathrm{HClO}}_{4}\;\overset{-OH,step\;A1}{\to }{\mathrm{ClO}}_{3}^{-}\;\overset{-O,step\;A2}{\to }{\mathrm{ClO}}_{2}^{-}\;\overset{-OH,step\;A3}{\to }{\mathrm{ClO}}^{-}\;\overset{-O,step\;A4}{\to }$$

**Figure 2:**
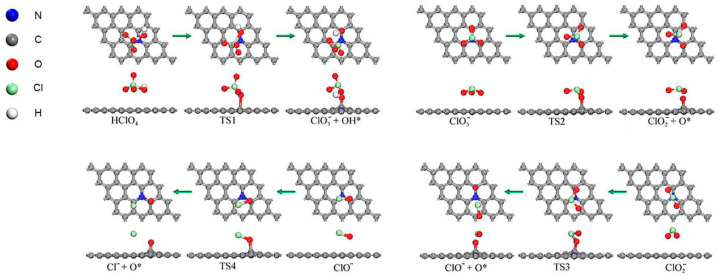
Optimized structures of decomposition reactions of PA and its intermediate products along the pathway that generated oxychloride on N-Gr surface, the symbol * indicates that the substance is chemisorbed on N-Gr and the green arrows indicate the reaction process for each elementary reaction.

**Figure 3:**
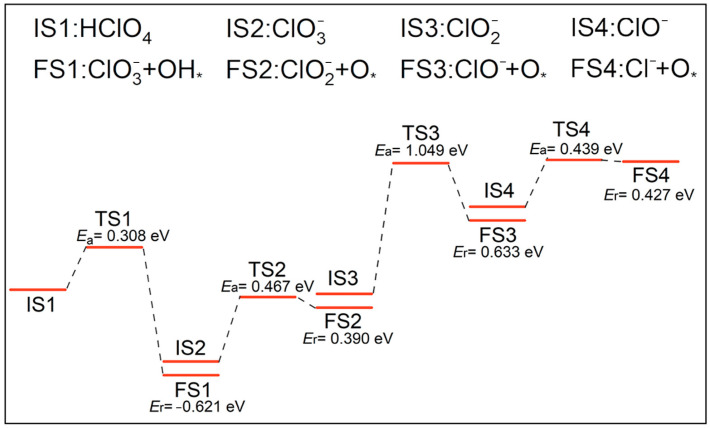
Energy profile of decomposition reactions along the chlorate route, the symbol * indicates that the substance is chemisorbed on N-Gr.

The initial state (IS), transition state (TS), and final state (FS) structures of these reactions are illustrated in Figure 2, and the energy profiles on the reaction paths are shown in Figure 3. When the HClO_4_ molecule decomposes on the clean N-Gr surface, HClO_4_ would firstly undergo a dehydroxylation reaction (A1) to produce ${\mathrm{ClO}}_{3}^{-}$ and hydroxyl groups. The dissociated hydroxyl groups would adsorb on the o-C atoms on the N-Gr surface. The activation energy of this reaction is 0.308 eV, and the heat of reaction was −0.621 eV, with negative values indicating that the reaction is exothermic. Subsequently, the newly generated ${\mathrm{ClO}}_{3}^{-}$ experienced a multi-step deoxygenation reaction to produce ${\mathrm{ClO}}_{2}^{-}$, ClO^−^, and Cl^−^ gradually. Moreover, this process produced a large number of O atoms which tended to adsorb on the N-Gr surface. The activation energies for the Cl-O bond-breaking reactions of ${\mathrm{ClO}}_{3}^{-}$, ${\mathrm{ClO}}_{2}^{-}$, and ClO^−^ on the clean N-Gr surface were 0.467, 1.049, and 0.439 eV, and the corresponding heats of reaction are 0.390, 0.633, and 0.427 eV. It is clear that the rate-determining step in the decomposition of route A is the deoxygenation of the ${\mathrm{ClO}}_{2}^{-}$.

**Figure 4:**
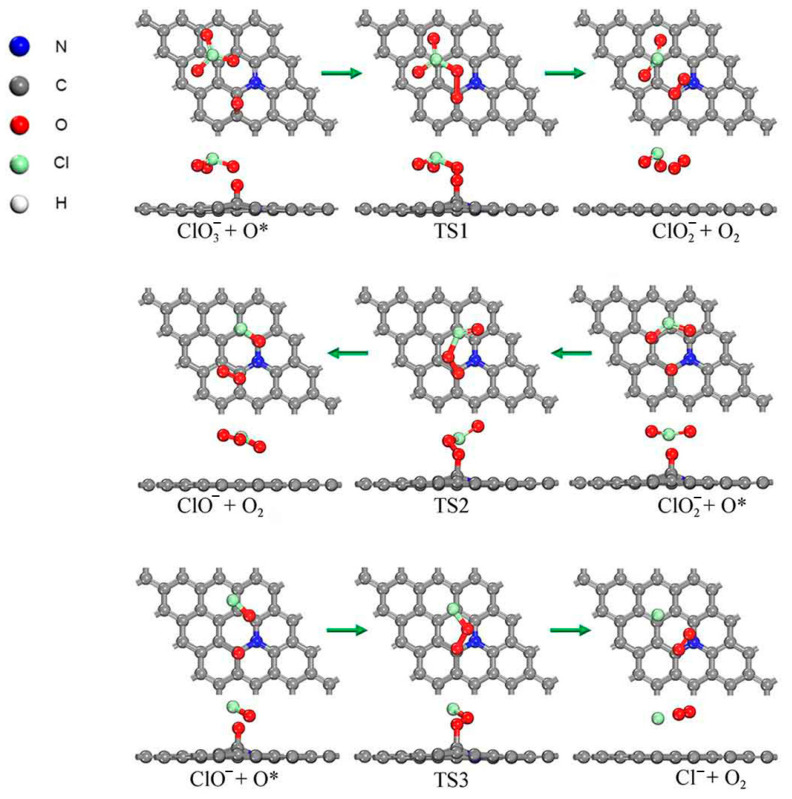
Optimized structures of decomposition reactions of intermediate products of PA along the pathway that generated oxychloride on oxidized N-Gr, the symbol * indicates that the substance is chemisorbed on N-Gr and the green arrows indicate the reaction process for each elementary reaction.

**Figure 5:**
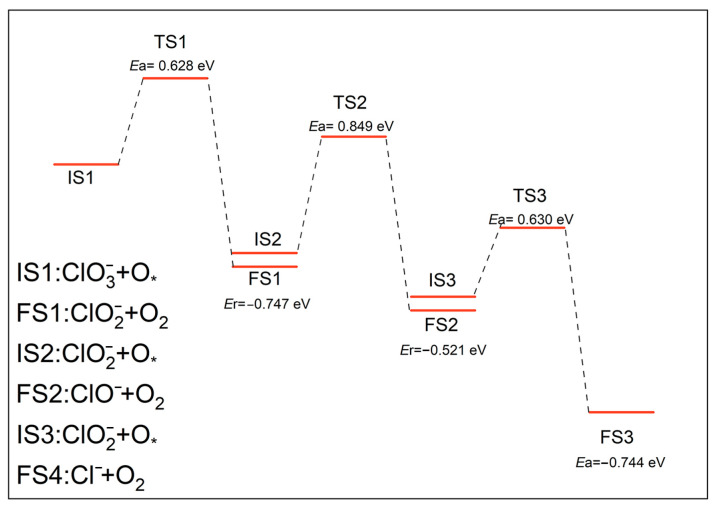
Energy profile of decomposition reactions of intermediate products of PA along the pathway that generated oxychloride on oxidized N-Gr, the symbol * indicates that the substance is chemisorbed on N-Gr.

In addition, it was noted that with the decomposition of HClO_4_, many O atoms may adsorb on the N-Gr surface. Therefore, the reaction of ${\mathrm{ClO}}_{x}^{-}$ on the oxidized N-Gr surface was investigated. The optimized structures and energy profiles of the reactions are shown in Figure 4 and Figure 5. It was found that the O atom of ${\mathrm{ClO}}_{x}^{-}$ would form an O-O bond with Oad. Afterward, form O_2_ and ${\mathrm{ClO}}_{x-1}^{-}$ were formed. The rate-determining step in this process is also the reaction of ${\mathrm{ClO}}_{2}^{-}$, with a barrier of 0.849 eV and heat of −0.521 eV. Compared to the same reaction on the pure N-Gr surface, the activation energy is reduced by 0.2 eV. And the oxygen molecules generated in each elementary reaction would desorb and enter into gas-phase. It indicates that the oxidation of the N-Gr surface will facilitate the decomposition of HClO_4_ molecules.

### 2.3. Reaction Pathway for the First Step of Losing O Atoms (Oxygenated Chlorate Pathways)

Some researchers intended to ascertain the decomposition path of AP by analyzing its decomposition products. It was concluded that, when Co_3_O_4_ or ZnO were used as catalysts, the HClO_2_ and HClO molecules were detected as products [27,28]. Therefore, the decomposition path was proposed in which HClO_4_ loses its O atoms and experiences decomposition. In this reaction path, HClO_4_ proceeds to successive Cl-O bond breaks until finally the HO-Cl bond breaks to form Cl^−^ and OH. This reaction path was noted as reaction route B, as shown below.

Route B:

$${\mathrm{HClO}}_{4}\;\overset{-O,step\;B1}{\to }{\mathrm{HClO}}_{3}\;\overset{-O,step\;B2}{\to }{\mathrm{HClO}}_{2}\;\overset{-O,step\;B3}{\to }\mathrm{HClO}\overset{-OH,step\;B4}{\to }$$

**Figure 6:**
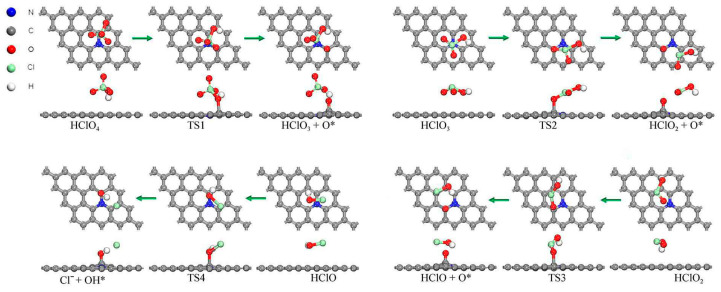
Optimized structures of decomposition reactions of PA along the oxygenated chlorate pathway, the symbol * indicates that the substance is chemisorbed on N-Gr and the green arrows indicate the reaction process for each elementary reaction.

**Figure 7:**
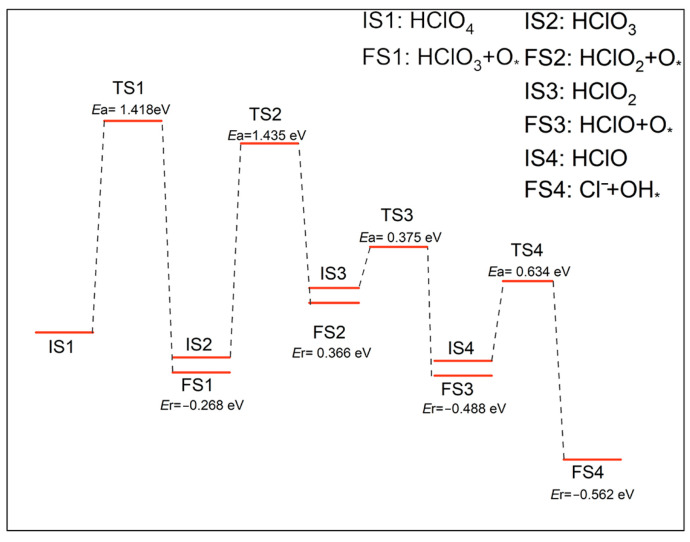
Energy profile of decomposition reactions of PA on N-Gr surface along the oxygenated acid route, the symbol * indicates that the substance is chemisorbed on N-Gr.

The optimized structure and the energy profile of the reaction were shown in Figure 6 and Figure 7. The decomposition of the HClO_4_ molecule on the N-Gr surface along route B occurred firstly by the break of the Cl-O bond to produce the HClO_3_ molecule and an O atom. The dissociated O atom would adsorb at the o-C atom top site by forming a C-O bond with the C atom. The reaction (B1) had an activation energy barrier of 1.418 eV and released −0.268 eV of energy. Subsequently, the HClO_3_ molecule experienced two deoxygenation reactions to decompose sequentially to form HClO_2_ and HClO molecules. The reaction (B2) for the decomposition of HClO_3_ to form HClO_2_ had activation energies and a heat of reaction of 1.425, and 0.366 eV and had the highest activation energy of all the reactions in this pathway. Thus, it is the rate-determining step of this path. The reaction potential and heat of reaction for step B3 were 0.375 and −0.488 eV. After this, the HClO molecule generated OH groups and Cl^−^ on the N-Gr surface, and the reaction was labeled as B4. The OH group adsorbed at the top site of the o-C atom, and Cl^−^ adsorbed by physisorption on the N-Gr surface through the formation of hydrogen bonding with the H atoms of the hydroxyl group. The activation energy and heat of the step B4 were 0.634 eV and −0.562 eV, respectively.

Compared with path A in the previous section, the activation energies of A1 and A2 were significantly lower than those of B1 and B2. This implied that, when the HClO_4_ molecule decomposes on the N-Gr surface, the acid-root reaction path (route A) is more likely to occur from thermodynamic considerations

### 2.4. Conversion Between Two Different Paths Above

**Figure 8:**
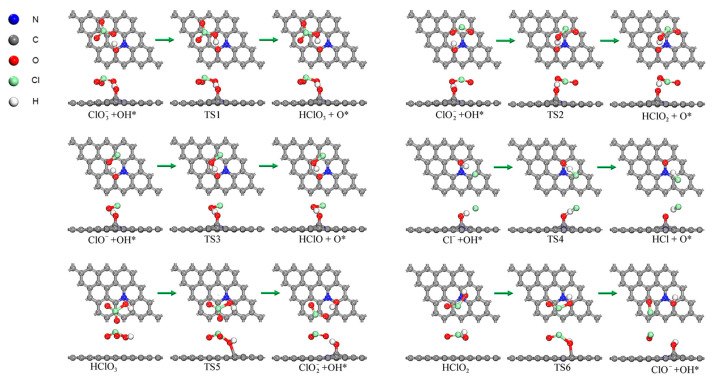
Optimized structures of possible reactions for mutual conversion between acid radical path and oxygenated acid path, the symbol * indicates that the substance is chemisorbed on N-Gr, the symbol * indicates chemisorption on N-Gr and the green arrows indicate the reaction process for each elementary reaction.

**Figure 9:**
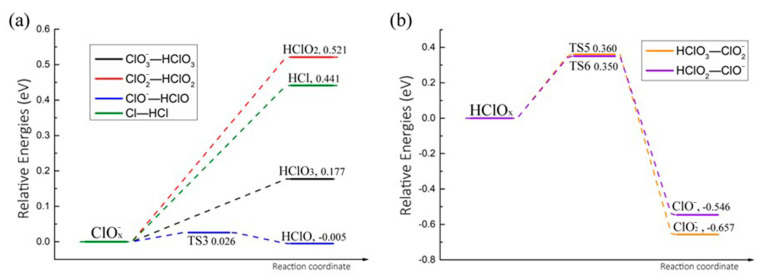
Energy profile of other possible decomposition reactions of intermediate products of PA on N-Gr expect the chlorate route and oxygenated acid route. (**a**) ${\mathrm{ClO}}_{x}^{-}$ groups react with OH to form HClO_x_ (**b**) HClO_x_ groups lose OH to form ${\mathrm{ClO}}_{x}^{-}$.

After discussing the adsorption of O atoms, the adsorption of OH groups on the N-Gr surface during the reaction is unavoidable. It is considered that the earliest OH groups may be generated by the decomposition of HClO_4_. In this case, the OH group may form the intermolecular hydrogen bonds with ${\mathrm{ClO}}_{3}^{-}$ thereby inhibiting the diffusion of ${\mathrm{ClO}}_{3}^{-}$. Thus, transition state calculations were performed for the reactions occurring between ${\mathrm{ClO}}_{x}^{-}$ (x = 1~3) and OHad. The corresponding reaction structures and energy profiles are shown in Figure 8 and Figure 9.

As shown in Figure 8, since the A1 reaction occurs after the adsorption of HClO_4_ molecules onto the N-Gr surface, the dissociated OH group was adsorbed onto N-Gr, where ${\mathrm{ClO}}_{3}^{-}$ formed a hydrogen bond with the adsorbed OH group. After that, H atoms would transfer to form HClO_3_ molecules. The reaction required overcoming an energy barrier of 0.151 eV and absorbing heat of 0.177 eV. The value of reaction heat was higher than the barrier, implying that the process is a heat-absorbing reaction with no barrier. Furthermore, the reactions of proton transfer reactions between ${\mathrm{ClO}}_{2}^{-}$, ClO^−^, and Cl^−^ and OHad groups were all the same as that of ${\mathrm{ClO}}_{3}^{-}$, in which the protonation reactions of ${\mathrm{ClO}}_{2}^{-}$ and Cl^−^ are also barrierless and heat-absorbing reactions. Frequency analysis and transition state confirmation also confirm the above conclusions. Additionally, the activation energy and heat of reaction for the reaction between ClO^−^ and OH_ad_ were 0.026 and −0.005 eV, respectively. Low values signify that the reverse reaction of this reaction is equally easy to occur.

Transition state searches were also carried out for the dehydroxylation of HClO_3_ and HClO_2_ molecules on the clean N-Gr surface. The optimized structures and the energy profiles of these reactions are shown in Figure 8 and Figure 9b. The Cl-O bonds of HClO_3_ and HClO_2_ molecules decompose to form hydroxyl groups, ${\mathrm{ClO}}_{2}^{-}$ and ClO^−^. And the OH groups were adsorbed at the top positions of o-C atoms. As to HClO_3_ and HClO_2_ molecules, activation energies of the above reactions were 0.360 and 0.350 eV, and the corresponding heats of reaction are −0.657 and −0.546 eV, respectively.

### 2.5. Mechanistic Analysis of the Decomposition Reaction of HClO_4_ on the N-Gr Surface

**Figure 10:**
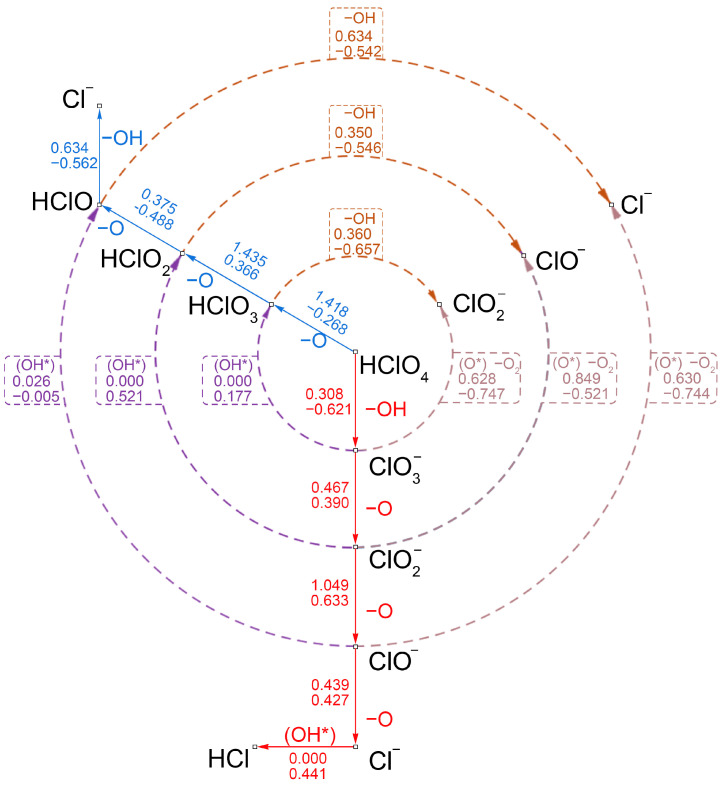
The reaction network of the decomposition of perchloric acid on the N-Gr surface, the symbol * indicates that the substance is chemisorbed on N-Gr, and the colorful dotted lines indicate transformations between different intermediates.

After discussing the different reaction paths, the decomposition mechanism of the HClO_4_ molecule on the N-Gr surface was determined through the reaction relationships of the individual intermediates, and the activation energies and reaction heats were listed. As shown in Figure 10, comparing the activation energies of the various reaction branches along the way, it is possible to determine the trends in the production and consumption of intermediates, thus clarifying the specific decomposition mechanism of HClO_4_.

As shown by the red arrows in Figure 10, when the HClO_4_ molecule decomposes along the acid-root decomposition path, OH groups and ${\mathrm{ClO}}_{3}^{-}$ were firstly produced. After this, ${\mathrm{ClO}}_{3}^{-}$ on the N-Gr surface gradually decomposed and generated ${\mathrm{ClO}}_{2}^{-}$, ClO^−^, and Cl^−^ and many O atoms. Furthermore, compared to other catalysts [33,45,46], energy barrier of the rate-determining step in AP decomposition is the lowest on N-Gr surface, which is 1.049 eV, lower than 1.595 eV (MgO) [33], 1.903 eV (g-C_3_N_4_) [45], and 2.071 eV (F-Gr) [46]. On the clean N-Gr surface, O atoms were adsorbed on the top sites of o-C atoms. Then, the O atom of the chlorine group forms an O-O bond with the absorbed O. With the complete breakage of the C-O_ad_ bond, the O_2_ molecules were generated which adsorbed on the N-Gr surface by physical adsorption. Then, O_2_ may be further desorbed into the gas phase.

In addition, the ${\mathrm{ClO}}_{3}^{-}$, generated by the decomposition of HClO_4_, experienced diffusion and deoxygenation on the clean N-Gr surface. Then, the ${\mathrm{ClO}}_{2}^{-}$ can directly react with the O atom, which just newly dissociated and adsorbed at the top site of o-C atom to form ClO^−^ and O_2_ molecules. And the ClO^−^ would undergo a Cl-O bond-breaking reaction to form O_ad_ and Cl atoms. In this case, the activation energy of the rate-determining step in the acid radical pathway was 0.849 eV (reaction between the ${\mathrm{ClO}}_{2}^{-}$ and O_ad_ atom). Meanwhile, the diffusion of intermediate products occurs little on the N-Gr surface. In contrast, O atoms dissociated from HClO_4_ molecules partly adsorb on the N-Gr surface as O atoms and OH groups and partly diffuse into the gas phase as O_2_ atoms.

**Figure 11:**
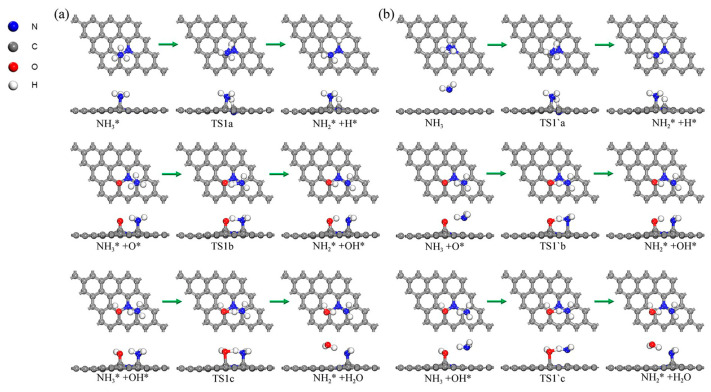
The optimized structures of dehydrogenation reactions of NH_3_ absorbed on N-Gr surfaces. (**a**) reaction structure of NH_3_ chemisorbed onto N-Gr; (**b**) reaction structure of NH_3_ physisorbed onto N-Gr, the symbol * indicates that the substance is chemisorbed on N-Gr. and the green arrows indicate the reaction process for each elementary reaction.

Along the oxygenated chlorate pathway (blue arrows in Figure 10), the HClO_4_ molecule gradually broke the Cl-O bond, which generated HClO_3_, HClO_2_, HClO molecules and Cl atoms in turn, and dissociated O and OH adsorbed on the N-Gr surface. However, the deoxygenation reaction of HClO_4_ molecules had a high reaction activation energy, so it was difficult for such molecules to decompose directly on the N-Gr surface. The purple arrows in Figure 11 showed the interconversion reactions between the intermediates of the two different pathways and their activation energies. The results showed that, even though the HClO_3_ molecule was generated at the N-Gr surface, it prefers to react directly with O atoms, adsorbing on o-C atoms, to form ${\mathrm{ClO}}_{3}^{-}$ and OHad molecules or to undergo dehydroxylation rather than deoxygenation along the oxygenated chloric acid pathway.

Although the energy barrier for the proton transfer reaction between ${\mathrm{ClO}}_{x}^{-}$ and OH_ad_ groups was lower than its activation energy for the Cl-O bond-breaking reaction, the inverse reaction of the proton transfer reaction between the ${\mathrm{ClO}}_{x}^{-}$ and the OH_ad_ group is spontaneous. Therefore, the HClO_x_ molecules were highly unstable. In summary, on the N-Gr surface, the HClO_4_ molecule decomposes mainly along the acid-root path.

### 2.6. Sequential Dehydrogenation of NH_3_ on the N-Gr Surface

Apart from HClO_4_, NH_3_ is also a primary product of AP decomposition. Meanwhile, it was mentioned that, with the decomposition of HClO_4_, O atoms and OH groups may be adsorbed on the catalyst surface [27,29]. So, this section discusses the decomposition process of NH_3_ on the N-Gr surface under different circumstances.

The adsorption of NH_3_ molecules on three different N-Gr surfaces (pure, adsorbed O, adsorbed OH) was studied. On the pure surface, the adsorption energies of NH_3_ carried out on the top sites of o, m, and p-C atoms were all positive. Among them, the smallest adsorption energy of 1.991 eV was observed for adsorption on the top site of the o-C atom. When the O atom adsorbed on the top site of the o-C atom, the NH_3_ molecule adsorbed on the top site of the other o-C atom with an adsorption energy of 0.879 eV. When the OH group existed, the NH_3_ molecule adsorbed on the same site with an adsorption energy of 1.674 eV. Clearly, the presence of Oad and OHad both promoted the adsorption of NH_3_ molecules on the N-Gr surface, but the adsorption energy remained positive, indicating that the chemisorption of NH_3_ molecules on the N-Gr surface is a heat-absorbing process.

After this, the decomposition reactions of NH_3_ molecules on different N-Gr surfaces were studied, and the IS, TS, and FS structures of each reaction are displayed in Figure 11, while the heats of reaction and activation energies are listed in Table 2.

When NH_3_ dehydrogenated on the N-Gr surface, the H atom formed a C-H bond with another o-C atom. The activation energy of this reaction was 0.922 eV, and the heat of reaction was 0.176 eV. When O atoms adsorbed on the surface of N-Gr, the dissociated H atoms formed an OH group with Oad, and the transition state search result of the reaction indicated that the reaction is an exothermic reaction with no barrier, and the heat of reaction is −0.699 eV. When the OH group is present, the H atoms produced from the decomposition of NH_3_ would form an H_2_O molecule with this group, and, at the same time, the C-O bond was broken, and the H_2_O molecules were adsorbed on the surface of N-Gr by physical adsorption. This process was also a spontaneous exothermic reaction with a heat of reaction of −1.915 eV. These results all indicated that both O atoms and OH groups could promote the adsorption and decomposition of NH_3_.

**Figure A4:**
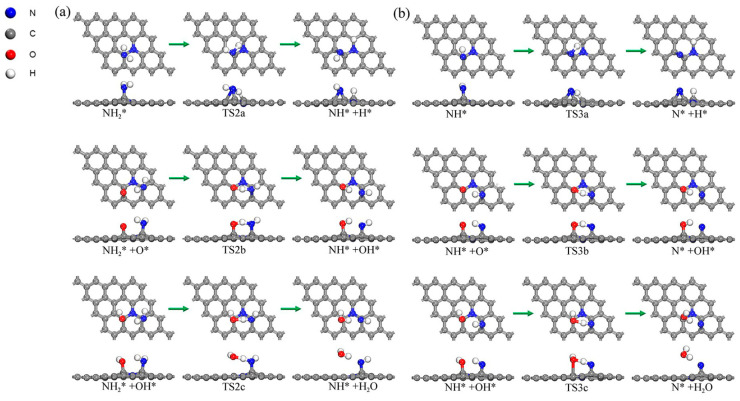
The optimized structures of dehydrogenation reactions of (**a**) NH_2_ (**b**) NH on N-Gr surfaces, the symbol * indicates that the substance is chemisorbed on N-Gr.

Considering that the chemisorption of NH_3_ groups occurring on different N-Gr surfaces were all heat-absorbing processes, the decomposition of NH_3_ groups during physisorption on different N-Gr surfaces needs to be taken into account. The structure of each radical reaction is shown in Figure A4b. As a result, NH_2_ decomposed from NH_3_ is chemisorbed onto o-C atoms on different surfaces. In addition, on pure N-Gr surfaces, H dissociated from NH_3_ molecules was likewise chemisorbed onto o-C atoms. It is a heat-absorbing reaction with an incompetent barrier, and its heat is 2.166 eV. When the O atom is present, H atoms bonded with it to form OH groups. The reaction had an activation energy of 0.472 eV and heat of 0.209 eV. In addition, when OH groups are present, H atoms combined to form H_2_O molecules and physically adsorbed on the N-Gr surface. The activation energy of this reaction was 1.101 eV, and the heat of reaction was −0.241 eV.

Regarding the decomposition reactions of NH_2_ and NH on the N-Gr surface, the structures of the radical reactions are shown in Figure A4, and the reaction heat data are also listed in Table 2. The results show that, when NH_2_ and NH decompose on the N-Gr surface, the generated NH and N atoms were transferred from the top position of the o-C atoms to the bridging positions of the o, m-C atoms, and formed a C-N bond with the m-C atom. When on the clean N-Gr surface, the dissociated H atoms were all adsorbed on another o-C atom, and the activation energies of the reactions of NH_2_ and NH decomposition were 4.114 and 1.955 eV, and the heat of reactions were 2.242 and 1.834 eV, respectively. However, when O atoms were present on the N-Gr surface, the H atom reacted with the Oad to form the OH group. The activation energies of the reaction were 0.770 and 0.175 eV, and the corresponding heat was 0.021 and 0.040 eV, respectively. Furthermore, NH_2_ and NH underwent a proton transfer reaction with OH groups adsorbed on the N-Gr surface and generated H_2_O molecules, and the activation energies of the reaction were 0.412 and 0.475 eV, and the heat was −0.039 and −0.178 eV, respectively.

**Figure 12:**
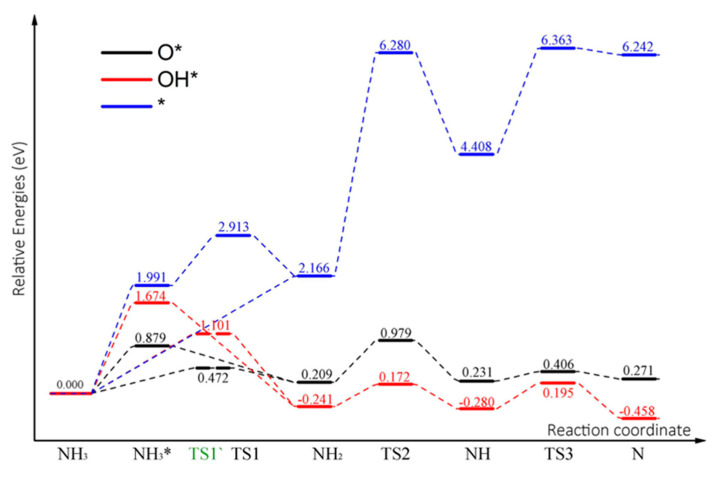
Energy profile of consecutive dehydrogenation reactions of NH_3_ molecules on N-Gr surfaces, the symbol * indicates that the substance is chemisorbed on N-Gr.

Figure 12 illustrates the reaction energy profiles for the decomposition of NH_3_ molecules on different N-Gr surfaces. More energy is required for NH_3_ molecules to undergo chemisorption on N-Gr surfaces. In the presence of O atoms and OH groups, the activation energy for the decomposition reaction of physically adsorbed NH_3_ to occur was lower compared to the energy for chemisorption. However, the activation energy for NH_3_ decomposition on the N-Gr surface after both pure and OH group adsorption was higher than the activation energy for decomposition along the acid-root pathway for HClO_4_ analysis. In the presence of O atoms, physically adsorbed NH_3_ could decompose rapidly, and the products adsorb on the N-Gr surface. Therefore, the presence of O_ad_ atoms can effectively promote the consumption of NH_3_ molecules. The dehydrogenation of NH_2_ and NH on the clean N-Gr surface was difficult to occur because of the high activation energy, but the continuous dehydrogenation of NH_3_ molecules on the N-Gr surface was possible in the presence of Oad or OHad. Also, the continuous dehydrogenation of NH_3_ molecules accelerates the O and OH adsorbed on the catalyst surface to detach from the catalyst surface, which promotes the reduction of the catalyst.

## 3. Materials and Methods

All calculations were carried out using the DMol^3^ module [47,48] of the Material Studio software (Version 8.0), which was developed using density functional theory. The generalized Perdew–Burke–Ernzerh (PBE) exchange-correlation potential from the generalized gradient approximation (GGA) [48] was chosen for the generalization to describe the electron exchange-correlation action in the system, which was proved to be applicable by our group [45,49,50] and some researchers [33,38]. The frequency calculations and the nudged elastic band (NEB) tool were used to search for stable geometries along the minimum energy pathway (MEP). Sampling k-points in the Brillouin zone was set to 4 × 4 × 1 for the N-Gr surface [49]. The van der Waals (vdW) interactions were made using the Grimme method (DFT-D3) [45]. The electronic occupancies were determined according to the Methfessel–Paxton scheme with an energy smearing of 0.001 Ha (1 Ha = 27.21 eV). Furthermore, the double numerical plus polarization (DNP) was employed to increase accuracy [51]. In order to search the stabilizing model accurately, the convergence criteria for energy, maximum force, maximum displacement and SCF on all atoms were set separately as 1.0 × 10^−5^ Ha, 0.002 Ha/Å, 0.005 Å and 1.0 × 10^−6^ Ha. The complete LST/QST approach (liner/quadratic synchronous transit) was employed to search the TS structure. And the activation energy (*E*_a_) and reaction energy (*E*_r_) of the redox reaction of AP and its decomposition products were calculated.

The formulation of *E*_a_, *E*_r_ and absorption energy (*E_ad_*) [50] are shown in Equations (1)–(3).

(1) $$E_{a}={\;E}_{TS}-E_{IS}$$

(2) $$E_{r}=E_{FS}-E_{IS}$$

(3) $$E_{ad}={\;E}_{B/surf}-(E_{surf}+E_{B})$$

where *E*_IS_, *E*_TS_, and *E*_FS_ are the total energies of the initial states (IS), TS, and the finial states (FS), respectively. *E_surf_* is the total energy of the N-Gr surface, *E_B_* is the energy of isolated adsorbate, and *E_B/surf_* is the total energy of the compound system.

## 4. Conclusions

In this study, density functional theory was employed to investigate the adsorption and decomposition processes of HClO_4_ and NH_3_ molecules on the N-Gr surface. In addition, the decomposition reaction mechanism of AP on N-Gr catalyst indication was described. The decomposition reaction path of HClO_4_ molecules on the N-Gr surface is proposed as HClO_4_ → ${\mathrm{ClO}}_{3}^{-}$ → ${\mathrm{ClO}}_{2}^{-}$ → ClO^−^ → Cl^−^. The rate-determining step of this process is the Cl-O bond-breaking reaction of the ${\mathrm{ClO}}_{2}^{-}$, and the activation energy of the reaction is 0.849 eV. Second, during the decomposition of the HClO_4_ molecule, the dissociated O atoms existed in two forms. One is that the chemisorption occurs on the N-Gr surface in the form of O atoms, the OH groups. The other one is in the form of O_2_ molecules that were physically adsorbed on the N-Gr surface and might desorb into the gas phase. The desorbed O_2_ molecules react with NH_3_ in the gas phase. And adsorbed O atoms and OH groups can promote the decomposition and consumption of NH_3_ molecules on the N-Gr surface.

This work helps to strengthen the understanding of the catalytic performance of N-Gr for AP combustion alone so as to better explain the catalytic mechanism of N-Gr. In addition, further study can be carried out to verify the thermal decomposition performance of N-Gr/AP through design experiments such as differential scanning calorimetry (DSC), thermogravimetry (TG) combined with FT-IR, MS, etc. More accurate theoretical calculation methods, such as newer DFT exchanges like revPBE, temperature correction [44], etc., are expected to be used to explain the decomposition process of AP on catalysts. Through the rational combination of experiment and theory, the catalytic decomposition effect of catalysis to AP can be better explained.

## Figures and Tables

**Table 1:** The adsorption energies and bond length of oxygen and hydroxyl group on N-Gr, the ^a^ indicates that O atoms adsorbed at the top site of m or p-C are transferred to the bridge site; O atoms adsorbed at the bridge site of o-C-N are transferred to the o-C top site. The ^b^ indicates that OH groups adsorbed at the top site of N are transferred to the o-C top site.

	Adsorption Sites	Ead/eV	d(C-O)/Å
O ^a^	Bridge, o, m-C	−3.225	1.437, 1.542
	Bridge, m, p-C	−2.823	1.487, 1.487
	Top, o-C	−3.442	1.318
	Top, N	−1.537	1.396
OH ^b^	Top, o-C	−2.393	1.457
	Top, m-C	−1.77	1.499
	Top, p-C	−1.969	1.494

**Table 2:** The activation energy (*E*_a_) and reaction heat (*E*_r_) of NH_3_ dehydrogenation on N-Gr surfaces (*E*_a_ and *E*_r_ in eV).

Pre-Absorbed Species	-		O Absorbed		OH Absorbed	
	*E*_a_	*E*_r_	*E*_a_	*E*_r_	*E*_a_	*E*_r_
NH_3_	-	2.166	0.472	0.209	1.101	−0.241
NH_3,ad_	0.922	0.176	-	−0.669	-	−1.915
NH_2,ad_	4.114	2.242	0.770	0.021	0.412	−0.039
NH_ad_	1.955	1.834	0.175	0.040	0.475	−0.178

**Figure A1:**
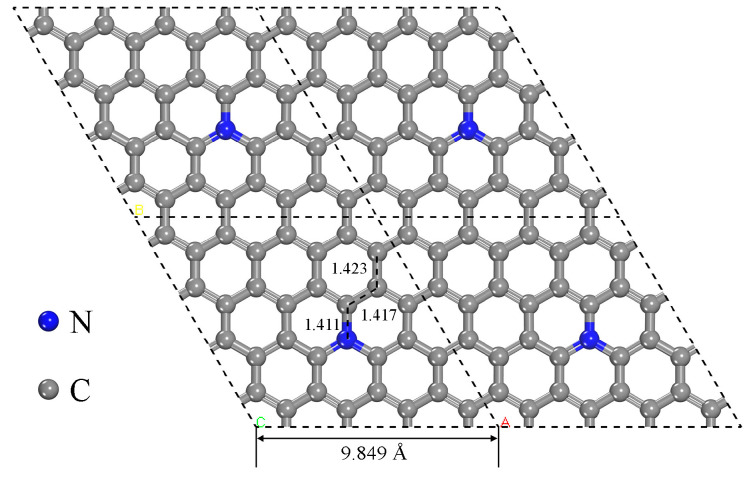
The supercell structure of single-layer N-doped graphene, colorful letters and dotted lines indicate the boundaries of the periodic structure.

**Figure A2:**
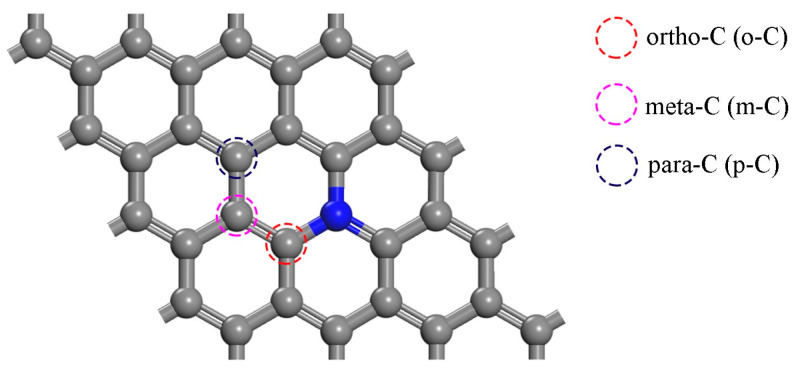
Three possible C atoms as adsorption sites, ortho-C (o-C), meta-C (m-C) and para (p-C).

## Data Availability

Data are contained within the article.

## Appendix A

### The Structure of N-Doped Graphene

The graphene surface is doped by replacing a C atom with a N atom. The geometry optimization was used to obtain the N-atom doped graphene surface as shown in Figure A1. The constructed N-Gr structure is a 4 × 4 supercell containing 31 C atoms and 1 N atom. And a vacuum layer of 15 Å in the *z*-axis direction was set up to prevent interlayer interactions on the N-Gr surface [20,33,45,46,47]. During the optimization, the cell was also optimized, and the cell parameters of the stabilized structure were calculated as: a = b = 9.849 Å, c = 19.172 Å, α = 89.95°, β = 89.72°, and γ = 120.08°. Compared to the intrinsic graphene, there is a slight enlargement of the supercell after doping with N atoms, which leads to a change in the C-C bond lengths on the N-Gr surface. The N-C bond length is 1.411 Å, and the o-C to m-C bond length to 1.417 Å, which is in general agreement with the value reported in the literature [37]. In contrast, the average bond length of p-C atoms as well as their outer C-C bond lengths elongated to 1.423 Å due to the expansion of the crystal cell.

In addition, the Mulliken population analysis of the three C atoms near the N atoms gives the results shown in Table A1. The results show that the C atom at o and p position is electron deficient, the ortho site (0.174 e) has more electron deficient charge than the para site (0.033 e). And the meta site (−0.007) shows little electron enrichment.

**Table A1:** The result of Mulliken population analysis.

Species	Mulliken Population Analysis (e)
o-C	0.174
m-C	−0.007
p-C	0.033
